# Supplementary material for: Rapid turnover of life-cycle-related genes in the brown algae
Source: Genome Biol. 2019 Feb 14;20:35. doi: 10.1186/s13059-019-1630-6 (PMC6374913; doi:10.1186/s13059-019-1630-6)
Supplement: Supplementary file 3 — Protein alignments, corresponding Gblocks html file and CDS sequences for the lineage conserved gametophyte-biased genes. (ZIP 2109 kb) [file 13059_2019_1630_MOESM3_ESM.zip › Con_GA_alignments/Ec-08_000340.aa_ali.fasta-gb.htm]

Ec-08\_000340.aa.fasta.trans.aa\_ali.fasta


## Gblocks 0.91b Results

Processed file: **Ec-08\_000340.aa.fasta.trans.aa\_ali.fasta**  
Number of sequences: **2**  
Alignment assumed to be: **Protein**  
New number of positions: **207** (selected positions are underlined in blue)

```
                         10        20        30        40        50        60
                 =========+=========+=========+=========+=========+=========+
Ec-08_000340     MVTCGLCGYRGPGEGTECAVCGQPHDRARDREQERSAAGSRPGQTNGGLWYEKPPQKSRI
MSTRG.49914.1    ------------------------------------------------------------
                                                                             


                         70        80        90       100       110       120
                 =========+=========+=========+=========+=========+=========+
Ec-08_000340     GAQAMSRCNSIPGGDRSTHLNPDLRYLLNLIPEQQNRRKRAEDVVRATGACLTPARADGD
MSTRG.49914.1    ------------------------------------------------------------
                                                                             


                        130       140       150       160       170       180
                 =========+=========+=========+=========+=========+=========+
Ec-08_000340     GCDPDVVYIRPRVDPTMRVLKGRYKTQPRIAKSTSMSALHRLADPLMGYDSIEALAPKER
MSTRG.49914.1    ------------------------------------------------------------
                                                                             


                        190       200       210       220       230       240
                 =========+=========+=========+=========+=========+=========+
Ec-08_000340     TLRRESVERRLGQALASGNPGGQGRLLNTTRPSRTNNTVLGRSSSGGSIPQQTPMAISVN
MSTRG.49914.1    ------------------------------------------------------------
                                                                             


                        250       260       270       280       290       300
                 =========+=========+=========+=========+=========+=========+
Ec-08_000340     AVSLQDRRQRSIGSTAVQQRRGYRPGSRWERSKTQEIAVSPLLRLRASGGRRVKPTLRRK
MSTRG.49914.1    ------------------------------------------------------------
                                                                             


                        310       320       330       340       350       360
                 =========+=========+=========+=========+=========+=========+
Ec-08_000340     RVATMRSVNGWVKEADPASMRRQNSLDRRFIALREKRNRKPKGKRKDSRPLAHSSGATAR
MSTRG.49914.1    ----MRSVNGWIRETDPTSRRQHMSLDRQIVALRKKGDR----HRRENHNLSDSAGANTQ
                     ###################################     #############   


                        370       380       390       400       410       420
                 =========+=========+=========+=========+=========+=========+
Ec-08_000340     -DSRAFDGSTFLTEVETDVVSVPVGGTRTSSGDRRNGGWVEDSQEQRQRKGGEANPARRH
MSTRG.49914.1    GVHVGGDSATFLTELETEG-SAPAAEINPSQGD--TTIYPEMADCQRRGRDERGSPGRKL
                       ###########   #############       ##################  


                        430       440       450       460       470       480
                 =========+=========+=========+=========+=========+=========+
Ec-08_000340     PGGRRRVSISGVARKPQTRGQLGRASGGASSTNTTRRRSTESDPAITGRGRSDVARRKGG
MSTRG.49914.1    ETNEKKASTMGVAYRGCRRRRSSGKGGAPFSTAHERRRLSDARLANARRDRSEVARFGNK
                        #####################################################


                        490       500       510       520       530       540
                 =========+=========+=========+=========+=========+=========+
Ec-08_000340     RTKSVGALNRFQVEKLNRGHVRASSLGRGGGDPKRPQPPPDNSGDLDMGRVVAADGASQW
MSTRG.49914.1    STKSIQVGN-HRNQQLREERYRTPAVAADIRVAER-------RRDVNVERRPEQLAQAII
                 #########                                                   


                        550       560       570       580       590       600
                 =========+=========+=========+=========+=========+=========+
Ec-08_000340     ALGNPGEAWTLSPSSRVDSNRVSQTTAELKLGVSTGDDTSAHGTSTTLAPAWGSPGSHSV
MSTRG.49914.1    SKGAGLEATKVVSAGHRAVVRRGGSTAAPAWGVSDSLAVSPTAEDSRVGRAWQGSGADTR
                                     ####################                    


                        610       620       630       640       650       660
                 =========+=========+=========+=========+=========+=========+
Ec-08_000340     GPLEARLTWQENEAGTSSRPCSEWQLLRRN-EKSSSRPAKMLHSFENKVG-SPLQLRQQH
MSTRG.49914.1    ---------------ALGQPCSEWQFLREGGCMSSFRSAELLSSFGNKVASSCLVDKQRR
                                                  ################  #########


                        670       680       690       700       710       720
                 =========+=========+=========+=========+=========+=========+
Ec-08_000340     PPTSVASNDEPAAQLLLSENSGPGDRWRPPDRSSPRRSDTKEPHPSESRLALMHHRPSSK
MSTRG.49914.1    RPTSAVANAE----------------KKPQQ-----------------------------
                 ##########                                                  


                        730       740       750       760       770       780
                 =========+=========+=========+=========+=========+=========+
Ec-08_000340     SWVHRRSLPSLLSNTTEPPSSLAGTSSTSTLTRGSPAAAAAGGGGAILRKRGRRRPSLDE
MSTRG.49914.1    -------------------------------------------------QRSPIFPSS--
                                                                             


                        790       800       810       820       830       840
                 =========+=========+=========+=========+=========+=========+
Ec-08_000340     PSGLMQQLDKNYAVTVGDTWGGGLSRRGAGKRRDEDDGGVEGVSAEAEAAGRGGTPSVVG
MSTRG.49914.1    ------------------------------------------------------------
                                                                             


                        850       860       870       880       890
                 =========+=========+=========+=========+=========+==
Ec-08_000340     CVGVGGDGATLTAAARPGSRGQGSTSSGRLLSSAIPKPLLYVGSGGGFRLRS
MSTRG.49914.1    ----------------------------------------------EEEEEX
```

```
Parameters used
Minimum Number Of Sequences For A Conserved Position: 2
Minimum Number Of Sequences For A Flanking Position: 2
Maximum Number Of Contiguous Nonconserved Positions: 8
Minimum Length Of A Block: 10
Allowed Gap Positions: None
Use Similarity Matrices: Yes
```

```
Flank positions of the 9 selected block(s)
Flanks: [305  339]  [345  357]  [367  377]  [381  393]  [401  418]  [428  489]  [561  580]  [634  649]  [652  670]  

New number of positions in Ec-08_000340.aa.fasta.trans.aa_ali.fasta-gb:  207  (23% of the original 892 positions)
```
